# False Detection of Cheyne–Stokes Respiration on Continuous Positive Airway Pressure Resolved After Treatment of Nasal Obstruction

**DOI:** 10.1002/rcr2.70745

**Published:** 2026-09-07

**Authors:** Emilie Castermans, Dimitri Impens, Walter Libert, Marie Bruyneel

**Affiliations:** ^1^ Department of Pulmonary Medicine Saint‐Pierre University Hospital Brussels Belgium; ^2^ Université Libre de Bruxelles Brussels Belgium

**Keywords:** central sleep apnea, Cheyne–Stokes respiration, continuous positive airway pressure, obstructive sleep apnea syndrome, telemonitoring

## Abstract

Cheyne–Stokes respiration (CSR) is a potentially dangerous abnormal breathing pattern that can be associated with serious cardiac events and poor prognosis. This case describes the unexpected recording of CSR events by a continuous positive airway pressure (CPAP) apparatus in a patient who was consistently CPAP compliant and had a stable medical history except for recent nasal obstruction. Respiratory polygraphy (RP) suggested the CPAP device had erroneously recorded obstructive sleep apnea (OSA) as CSR due to mixed apnea features that mimicked CSR. Treatment of the nasal obstruction resolved the problem with the erroneous readings, suggesting the nasal obstruction was the cause of the aberrant CPAP recording. This case highlights the limits of CPAP device reliability for detection of CSR and the need for standardized CSR detection criteria across CPAP devices.

## Graphical abstract

This case describes the unexpected recording of Cheyne–Stokes respiration (CSR) events by CPAP in a patient with recent onset of nasal obstruction. Respiratory polygraphy suggested the CPAP device had erroneously recorded OSA as CSR due to mixed apnea features that mimicked CSR. This case highlights the limits of CPAP device reliability for detection of CSR.

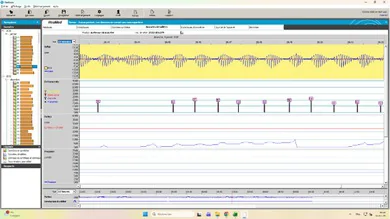

## 1 Introduction

When Cheyne–Stokes respiration (CSR) is detected by continuous positive airway pressure (CPAP), medical work‐up is mandatory as CSR can be associated with heart failure, arrhythmias, serious cardiac events and poor prognosis [1, 2]. The first step is to confirm CSR; the gold standard for its diagnosis is fully attended polysomnography (PSG) according to AASM criteria [3]. Respiratory polygraphy (RP) may detect a periodic breathing pattern suggestive of CSR, but lacks EEG and precise sleep staging, limiting its ability to definitively characterize central events and cyclic breathing [4]. Therefore, RP should be considered a screening tool, whereas PSG remains necessary for definitive diagnosis and therapeutic decision making. In the case of CSR detected by a CPAP device, RP is an acceptable first screening option and should include a microphone to facilitate the distinction between obstructive and central hypopneas [5].

## 2 Case Report

**FIGURE 1:**
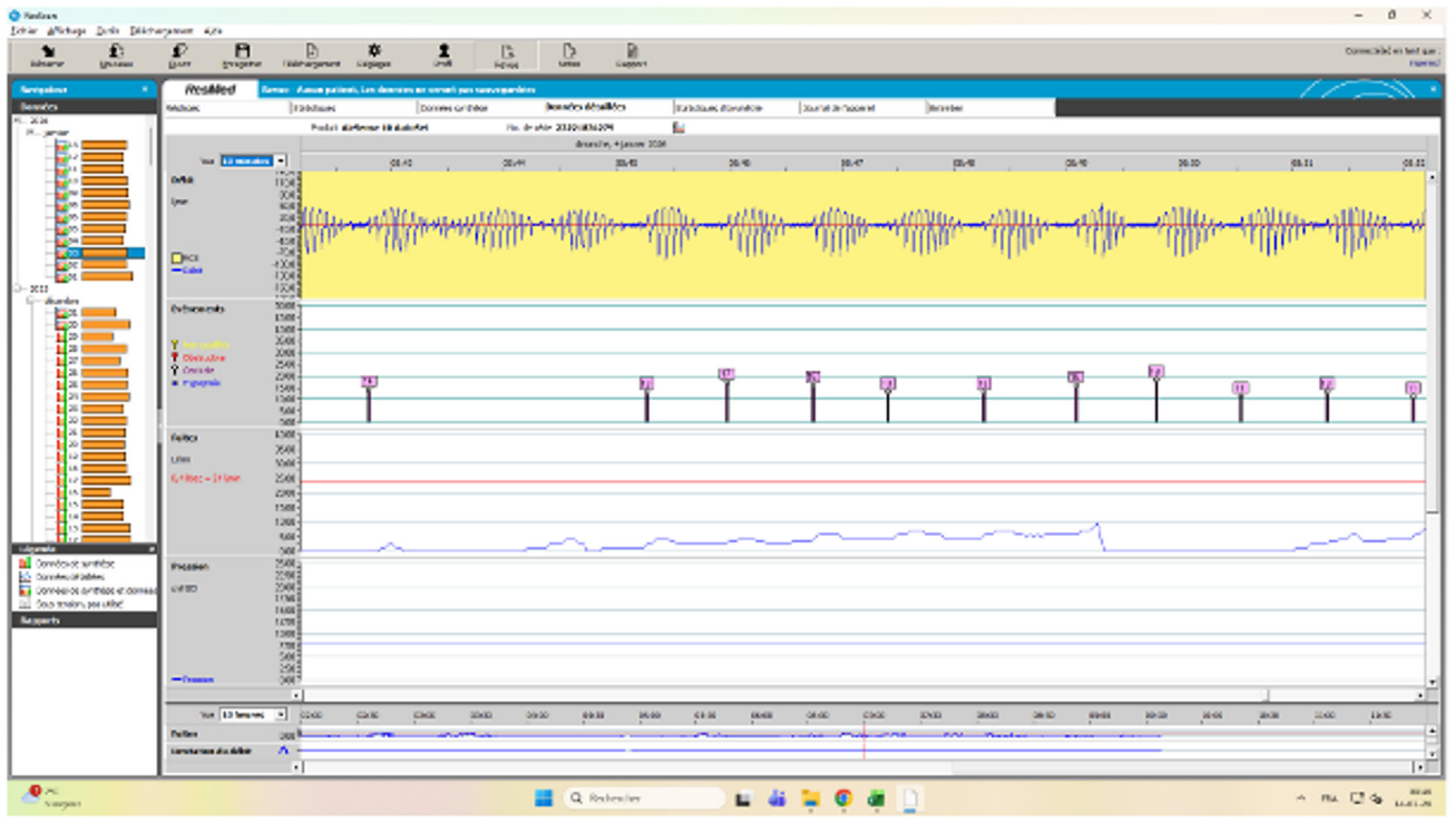
Ten minutes of fixed CPAP data extraction (night of 3 January, downloaded from the SD card of the Resmed Airsense10 device) showing Cheyne–Stokes respiration on the flow signal (highlighted in yellow) and central sleep apneas. Pressure level was 7.6 mbar.

A 56‐years‐old non‐obese consistent CPAP user observed the occurrence of residual respiratory events (RREs) recorded on his device on several consecutive mornings. When the data were downloaded from the SD card of the Resmed Airsense10 device, the unexpected occurrence of central sleep apnea was observed (total apnea‐hypopnea index (AHI_flow_) 8.7/h, central apneas 8.5/h and CSR 11%) (Figure 1). The leak level was low (median: 1.2 L/min, 95th percentile: 8 L/min). The patient had a non‐remarkable medical history and had a recent normal neurologic and cardiac work‐up, including brain MRI, cardiac echography and ergospirometry. His only current medication was aspirin.

The patient had been using a CPAP device for OSA syndrome for 13 years, at a fixed pressure of 7.6 mbar, with a nasal mask. His weight was stable, but he had recently developed nasal obstruction.

**FIGURE 2:**
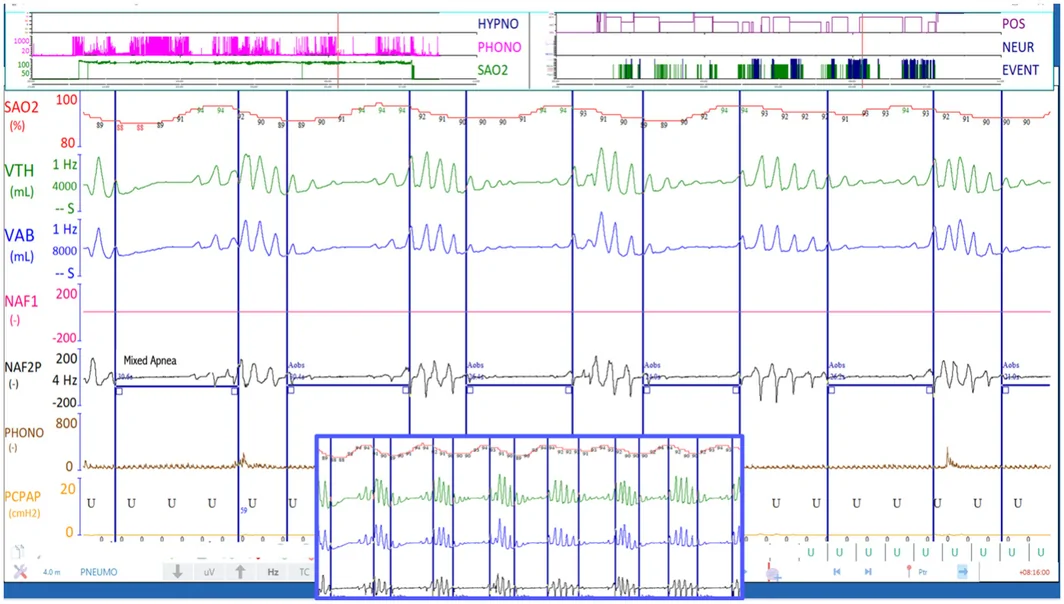
Respiratory polygraphy recorded with Dreamscan, Medatec, Belgium. On this 4‐min reading, respiratory signals showed mixed and obstructive apneas associated with snoring. The bottom image is a 10‐min reading extract, showing respiratory signal patterns that can mimic Cheyne–Stokes respiration despite large predominance of obstructive apneas and hypopneas associated with snoring. SA02: Oxygen saturation, VTH: Thoracic belt, VAB: Abdominal belt, NAF2P: Nasal pressure canula, PHONO: Microphone, PCPAP: CPAP pressure.

As CSR onset was unexpected in this situation, we performed a RP after 5 days of CPAP withdrawal. A clear OSA pattern was observed, including obstructive apnea and hypopnea associated with snoring. During this 659‐min recording, the total AHI was 27.8/h, the obstructive AHI was 21.8/h and the central AHI was 6.5/h. The oxygen desaturation index was 29.3/h. No CSR episodes were recorded. However, the pattern mimicked CSR as there were several mixed apneas with an initial central component (Figure 2). We hypothesized that the CSR detection by the CPAP device was erroneous. The CPAP pressure was progressively increased to 11 mbar, with instauration of a naso‐buccal mask to bypass nasal obstruction. The problem partially improved: no further CSR was detected but AHI_flow_ remained at 11/h. In the meantime, the patient received nasal corticosteroids for nasal obstruction, and 1 month later, with the same settings, AHI_flow_ decreased to < 5/h.

## 3 Discussion

In this patient case, false onset of CSR triggered by nasal obstruction in a consistent CPAP user was observed. CSR was not confirmed by RP. Increase in CPAP pressure and shift to naso‐buccal mask alleviated the CSR but RREs persisted until complete resolution of the nasal obstruction.

The criteria and algorithms for diagnosing CSR from a CPAP device have not been clarified for each CPAP manufacturer. For example, the Resmed Airsense10 CPAP device uses a forced oscillation technique. When the flow falls, if oscillations dissipate, it means that the upper airways are open, reflecting a central event. CSR detection is based on the recognition of a specific flow pattern, including crescendo‐decrescendo cycle length, duration of the episodes and presence of central events.

Respiratory control instability is also a factor that overlaps with CSR in patients with OSA. Loop gain (LG) is one of the four pathophysiological mechanisms implicated in OSA and includes three components: the control, the exchange and the connection component. LG > 1 reflects a (hyper‐)sensitive system leading to excessive ventilatory response (periodic breathing or CSR), whereas LG < 1 is associated with a more stable ventilatory system. A high LG in OSA, in non‐rapid eye movement sleep, can thus mimic CSR, at least for CPAP device algorithms.

Prigent et al. [1] reported in the AlertApnee study that CSR onset detection was attributable to obstructive events under CPAP when obstructive events occurred nearly systematically prior to CSR or when there was no criterion for alternative causes of CSR. They also observed recurrent typical obstructive apnea misclassified by the software as CSR in patients treated with the Resmed Airsense10.

In this case, after documentation of respiratory patterns by RP, the CSR turned out to be recent onset mixed sleep apnea. The transition from purely obstructive to mixed events appeared to be related to ventilatory control instability (high LG) triggered by nasal obstruction.

Nasal obstruction may increase ventilatory instability by elevating LG and promoting arousal‐related hyperventilation, thereby facilitating the occurrence of mixed apneas. This does not represent a phenotypic conversion of OSA into central sleep apnea but rather a secondary destabilization of ventilatory control. Indeed, nasal obstruction contributes to increasing inspiratory effort, leading to more frequent arousals and larger ventilatory overshoot after arousal. Altogether, this leads to unstable ventilatory control and mimics CSR.

To conclude, this case illustrates CSR detected by the CPAP device that was considered artifactual, likely related to nasal obstruction, ventilatory instability and pseudo‐periodic airflow patterns on CPAP flow analysis. Device‐based algorithms relying solely on airflow are known to misclassify obstructive or unstable breathing as CSR in the absence of respiratory effort signals. In the future, efforts should be made to improve the accuracy of CSR detection and to standardize criteria for determining CSR detected by CPAP devices from different manufacturers. Physicians should be aware of the limits of CPAP data collection reliability.

## Author Contributions

**Emilie Castermans:** investigation, validation, data curation, writing – original draft preparation, writing – reviewing and editing. **Dimitri Impens:** conceptualization, methodology, formal analysis, writing – reviewing and editing. **Walter Libert:** investigation, data curation, resources, writing – reviewing and editing. **Marie Bruyneel:** conceptualization, methodology, investigation, validation, formal analysis, supervision, data curation, writing – original draft preparation, writing – reviewing and editing.

## Funding

The authors have nothing to report.

## Consent

The authors declare that written informed consent was obtained for the publication of this manuscript and accompanying images using the consent form provided by the Journal.

## Conflicts of Interest

The authors declare no conflicts of interest.

## Data Availability

The data that support the findings of this study are available from the corresponding author upon reasonable request.
